# Execution, imitation and observation of naturalistic actions in autistic children and adolescents: a systematic review of fMRI studies

**DOI:** 10.3389/fnhum.2026.1786807

**Published:** 2026-04-13

**Authors:** Hanna Hjärtström, Anna-Maria Johansson, Sara Stillesjö, Thomas Rudolfsson, Daniel Säfström, Erik Domellöf

**Affiliations:** 1 Department of Psychology, Umeå University, Umeå, Sweden; 2 Umeå Center for Functional Brain Imaging (UFBI), Umeå University, Umeå, Sweden; 3 Department of Health, Education and Technology, Luleå University of Technology, Luleå, Sweden; 4 Department of Occupational Health, Psychology and Sports Sciences, University of Gävle, Gävle, Sweden; 5 Department of Medical and Translational Biology, Umeå University, Umeå, Sweden

**Keywords:** action execution, adolescents, autism spectrum disorder, children, fMRI, imitation, motor, observation

## Abstract

**Introduction:**

Motor difficulties are frequent in autistic children and associated with diverse social behavior, possibly due to atypical neural processing subserving internal action models. This systematic review synthesizes results from current functional magnetic resonance imaging (fMRI) research of brain activation during execution, imitation and observation of naturalistic actions in autistic children and adolescents (<18 years).

**Methods:**

Peer-reviewed articles in English published between 2000 and 2025 reporting task-related fMRI in diagnosed autistic vs. typically developing youth (<18 years) were evaluated. Eight studies (with a total of 129 autistic and 128 typically developing participants) were identified, divided into action execution (*n* = 1), observation (*n* = 4), and imitation (*n* = 3).

**Results:**

Between-group differences included reduced cerebellar activations for execution in autistic children; higher activity in left-lateralized motor processing regions for imitation; and lower activity in temporoparietal, posterior cingulate and anterior prefrontal cortex for observation.

**Discussion:**

Findings suggest that atypical brain activation during action execution, observation and imitation in autistic youth is frequent and largely support the notion of aberrant formation and use of motor representations in autism development. Although, due to the limited number of studies, small samples, variability in fMRI pipelines, and task specific nature of the results, interpretations require caution and further investigations are warranted.

## Introduction

Atypical motor performance and motor impairments are commonly observed in autistic individuals (Gowen and Hamilton, 2013) and have been reported at all ages, from infancy (Hadders-Algra, 2022) across childhood (Coll et al., 2020), adolescence (Bhat, 2023) and into adulthood (Fournier et al., 2010; Gowen et al., 2023). There are two broad categories of diagnostic criteria for autism spectrum disorder (ASD, autism): atypical social communication and interaction, and restrictive or repetitive behaviors, including atypical sensory processing (American Psychiatric Association, 2013). Although motor impairments are not included as diagnostic criteria, it has been estimated that 50–88% of children with an autism diagnosis have significant motor impairments (assessed by standardized tests or questionnaires) in functional motor performance, fine motor skills, and/or gross motor skills (Kangarani-Farahani et al., 2024). Impairments in motor performance related to ASD are also evident in studies using detailed kinematic measures, for example seen as reduced motor anticipation (Bäckström et al., 2021); poorer feedforward/feedback control of arm movements (Su et al., 2024); and less stability in motor plans associated with handwriting (Johnson et al., 2013).

The underlying mechanisms behind motor difficulties in autism remain largely unclear. There are, however, theoretical frameworks that seem to accommodate the idea that developing motor difficulties might be linked to the main symptoms of ASD, that is, social challenges (Mostofsky and Ewen, 2011), and atypical sensory processing (Falck-Ytter and Bussu, 2023). Mostofsky and Ewen (2011) suggest that atypical development and utilization of internal action models may contribute to both motor and social difficulties in autism. Internal action models are feed-forward simulations of discrete action plans and their resulting sensory feedback, applicable both to actions performed by oneself and by others. These models are thus essential for both successful execution of motor actions, as well as for motor-based understanding of others’ actions. Neurobiologically, this processing can be attributed to the action observation network (AON; e.g., Kilroy et al., 2019) and/or the mirror neuron system (MNS; e.g., Hamilton, 2013), together with the cerebellum (e.g., Wolpert et al., 1998; Streng et al., 2022). Another emerging theoretical framework endorsing ‘sensory-first’ accounts of autism (Falck-Ytter and Bussu, 2023; Robertson and Baron-Cohen, 2017) posits that early-life atypical sensory processing (evident in infants that are later diagnosed with autism; Falck-Ytter and Bussu, 2023) fundamentally impacts how the child interacts with and learns from the environment. In turn, this may affect other developmental domains (e.g., social, cognitive) and contribute to later emergence of autistic symptoms. Both these approaches (Falck-Ytter and Bussu, 2023; Mostofsky and Ewen, 2011) align with the well-established notion that typical development of motor, social, and sensory abilities occur in an interconnected and interdependent manner (e.g., Thelen and Smith, 1994; von Hofsten, 2004). For example, in the classic series of ‘visual cliff’ experiments (Gibson and Walk, 1960; Walk and Gibson, 1961) it was demonstrated that typical development of locomotive behaviors in infants relies on the development of visual processing, but also the other way around: depth perception does not fully develop without movement. Research has continued to shed light on the interrelatedness of early (typical) development of, for example, motor control and executive functioning (Gottwald et al., 2016); and motor skill proficiency and cognitive development (Capio et al., 2024). Studies investigating sensorimotor development in autism are sparse, however a recent longitudinal study shows atypical development of visuo-motor integration in autistic compared to typically developing (TD) children (Bäckström et al., 2025). Taken together, the importance of a developmental approach when researching sensorimotor challenges related to autism is evident.

Scientific interest in motor deficits and their possible association with atypical sensory and social processing in autism has increased significantly in recent years. Still, there is a shortfall of studies focusing on the neural processing behind atypical sensorimotor functioning in autistic individuals. Functional magnetic resonance imaging (fMRI) is the brain imaging technique that provides the most detailed measures of neural processing related to specific tasks or stimuli. Because of the high spatial resolution of the technique, it is well-suited both for exploratory research questions (e.g., comparing whole-brain activation), as well as for targeted comparisons (e.g., in terms of specific brain areas). However, not everyone can participate in fMRI experiments, due to for example sensory issues, claustrophobia, difficulties being still for a long period of time, or contraindications such as certain medical implants. For this reason, some populations (e.g., children, especially children with sensory issues such as in ASD) are more challenging to study with fMRI. Furthermore, studying brain activation related to movement execution using fMRI is another challenge since the technique is sensitive to head motion. Therefore, reviewing and synthesizing results from such studies with autistic participants are valuable means to increase knowledge of sensorimotor functioning in this condition.

In a systematic review of fMRI studies focusing on action execution and action observation in autistic compared to neurotypical (NT) adults, we concluded that the ASD groups showed atypical recruitment of brain regions associated with motor planning and/or feedforward control both when performing and when observing actions. For example, several studies found atypical recruitment of the bilateral inferior frontal gyrus (IFG: Müller et al., 2001, 2004; Grèzes et al., 2009; Okamoto et al., 2014) during both execution and observation of manual motor tasks in autistic adults. This region is important for motor processing and thus a brain region of interest in relation to atypical motor behavior in ASD. In addition, atypical activity in both the bilateral intraparietal cortices and the cerebellum may indicate difficulties in accessing stored motor representations, sensory motor integration as well as understanding actions of others (Cole et al., 2019; Grèzes et al., 2009; Lepping et al., 2022; Marsh and Hamilton, 2011; Müller et al., 2003; Okamoto et al., 2014). Evidently, motor difficulties in autism persist into adulthood and appear to be subserved by atypical brain activation patterns. Thus, from a developmental perspective, it is critical to learn whether atypical sensorimotor action in children with ASD is paralleled by similar or alternative atypical neural activity related to the interplay between perception and action and/or the MNS/AON. This will help to inform relevant interventions to optimize development and health in young autistic individuals. The aim of this review is to summarize the current knowledge of brain activation from fMRI studies focusing on aspects of ongoing motor processing associated with naturalistic actions in autistic children and adolescents (i.e., not including studies associating, e.g., behavioral outcomes with resting state fMRI). Here, we define ‘naturalistic actions’ as ordinary motor behavior in the physical world, for example direct use of gestures or interaction with physical objects (excluding, e.g., observation of cartoons, still images, point-light displays of movement, or performing actions in a digital environment).

The main interest is to compile brain regions where group differences between autistic children/adolescents (henceforth referred to as ‘ASD groups’) and children following typical development (TD groups) have been found during motor processing in fMRI paradigms. To further explore group differences in brain activation, we will also investigate qualitative aspects of within-group activations (i.e., signal strength and/or cluster size of activation) in brain areas showing group effects. Findings from our previous literature review focusing on autistic adults (Stillesjö et al., 2025) showed that ASD and NT groups recruited similar brain regions, but with group differences in the magnitude of brain activity during both action observation and action execution/imitation. Based on this, we anticipate that similar brain regions are recruited in children with ASD and TD while differences in activation patterns (direction and/or magnitude) are expected, as motor deviances are manifested early in life in ASD.

Furthermore, we will investigate if different domains of motor processing (i.e., action execution, observation, imitation) activate the AON and/or MNS similarly in ASD and TD children. Given findings from Stillesjö et al. (2025), we expect both action observation and imitation studies to find differences in brain regions associated with the MNS/AON (IFG in particular; see Müller et al., 2001, 2004; Grèzes et al., 2009; Okamoto et al., 2014).

## Methods

In this systematic review, we followed the Preferred Reporting Items for Systematic Review and Meta-Analysis (PRISMA) guidelines for reporting (Page et al., 2021). In adherence with the PICO/PECO framework, the population (P) consists of children/adolescents with autism, the intervention/exposure (I/E) is motor processing engagement, the comparison (C) is TD children/adolescents, and the outcomes (O) are brain activation patterns as measured by fMRI.

### Inclusion/exclusion criteria

Included articles had to be original research papers reporting task-related fMRI results in young (<18 years old) ASD vs. TD participants. Where age ranges were not reported, the mean sample age was used. Participants with ASD had to have a formal diagnosis following criteria in DSM-V or comparable. Furthermore, the studies had to include an experimental task focusing on execution, observation, or imitation of naturalistic motor actions with corresponding brain imaging results.

Articles were excluded if participants with ASD had been grouped with participants with other conditions (e.g., intellectual disability, developmental coordination disorder). Brain imaging articles with motor tasks that exclusively focused on socio-emotional aspects (e.g., imitation of facial expressions) or language (e.g., speech production) were also excluded, as were single-case studies.

### Search strategy

We searched PubMed, Web of Science, and Scopus for studies that had been published in peer-reviewed journals during the period between year 2000 – December 2025 and were written in English. The search string was designed as (P) AND (O) and (E), hence, the search was specified as “(Autism OR Asperger) AND (fMRI OR “functional magnetic resonance imaging”) AND (Action OR Movement OR Motor OR premotor).”

### Selection process

First, two authors (HH, SS) initially removed duplicates and records that could be marked as out of scope by screening the title. Next, all authors independently screened both the title and abstracts of an allocated set of the identified records by applying the inclusion/exclusion criteria. For reliability purposes, a second round of screening of titles and abstracts was performed where authors screened each other’s sets. Thereafter, all authors met to discuss potential remaining uncertainty related to inclusion/exclusion, where a decision for each publication was reached by consensus. The selected articles eligible for possible inclusion were then full text reviewed by the authors, applying exclusion criteria. Additional screening of records (including reference lists of the included articles) was performed independently by the authors where the same inclusion/exclusion criteria were applied. The review process of the final full text articles related to data extraction and analysis was performed by two authors (HH, AMJ).

### Risk of bias assessment

The quality of the included studies was evaluated using an adapted version of established approaches previously applied in review articles and meta-analyses of MRI research (Gentili et al., 2019; Iwabuchi et al., 2015; Kang, 2023) yielding a maximum score of 29. These approaches are largely based on the Newcastle-Ottawa scale (Wells et al., 2000) which was also applied in the present review with additional guidance from Stang (2010). Two evaluation items were added due to their particular relevance for this review: “A baseline was used and clearly defined” and “Comparison conditions/contrasts were clearly defined.” Quality assessment of Sample characteristics (max score = 7), Recruitment/selection bias (max score = 4), and Reproducibility/comparability (max score = 3) was conducted independently by authors AMJ and HH, while DS and SS independently evaluated the quality of Methodology and reporting (max score = 15). Any discrepancies between reviewers were resolved through discussion until consensus was reached. Details of the assessed quality of the studies and scoring can be found in Supplementary Table S1.

### Data extraction and analysis

Data extraction was performed independently and systematically from each individual article. Results and experimental protocols were extracted from the articles’ main result and method sections and synthesized based on the studies’ domain of focus (i.e., action execution, observation, or imitation). We extracted functional localization of blood-oxygenation-level-dependent (BOLD) peak activation in reported significant clusters for ASD vs. TD groups. Main outcome was significant group differences for the respective task domains (primarily experimental condition vs. baseline, but also within-group effects where between-group effects were found). Moreover, where available, measures of motor performance of the fMRI task, or other evaluations of motor ability that were correlated with brain activations, were also extracted. First, all results reported as Montreal Neurological Institute (MNI) or Talairach coordinates were transformed into Brodmann Areas (BA) using BioImage Suite Web^1^ (Papademetris et al., 2006). This step was carried out to synthesize all findings into one common system, despite the use of different T1 templates and coordinate atlases between studies. Next, all significant group effects in terms of brain activation were compiled and ordered based on BA, resulting in a synthesis of domain-overlapping and domain-specific activity, as well as a ‘mask’ of brain areas that seem relevant for motor processing. To gain more detailed information about brain activation patterns associated with this type of processing, we applied the mask to significant within-group activations (experimental condition > baseline for TD and ASD groups separately), based on available data regarding effect size and/or cluster size of the activation in all included studies.

## Results

### Study selection

**Figure 1:**
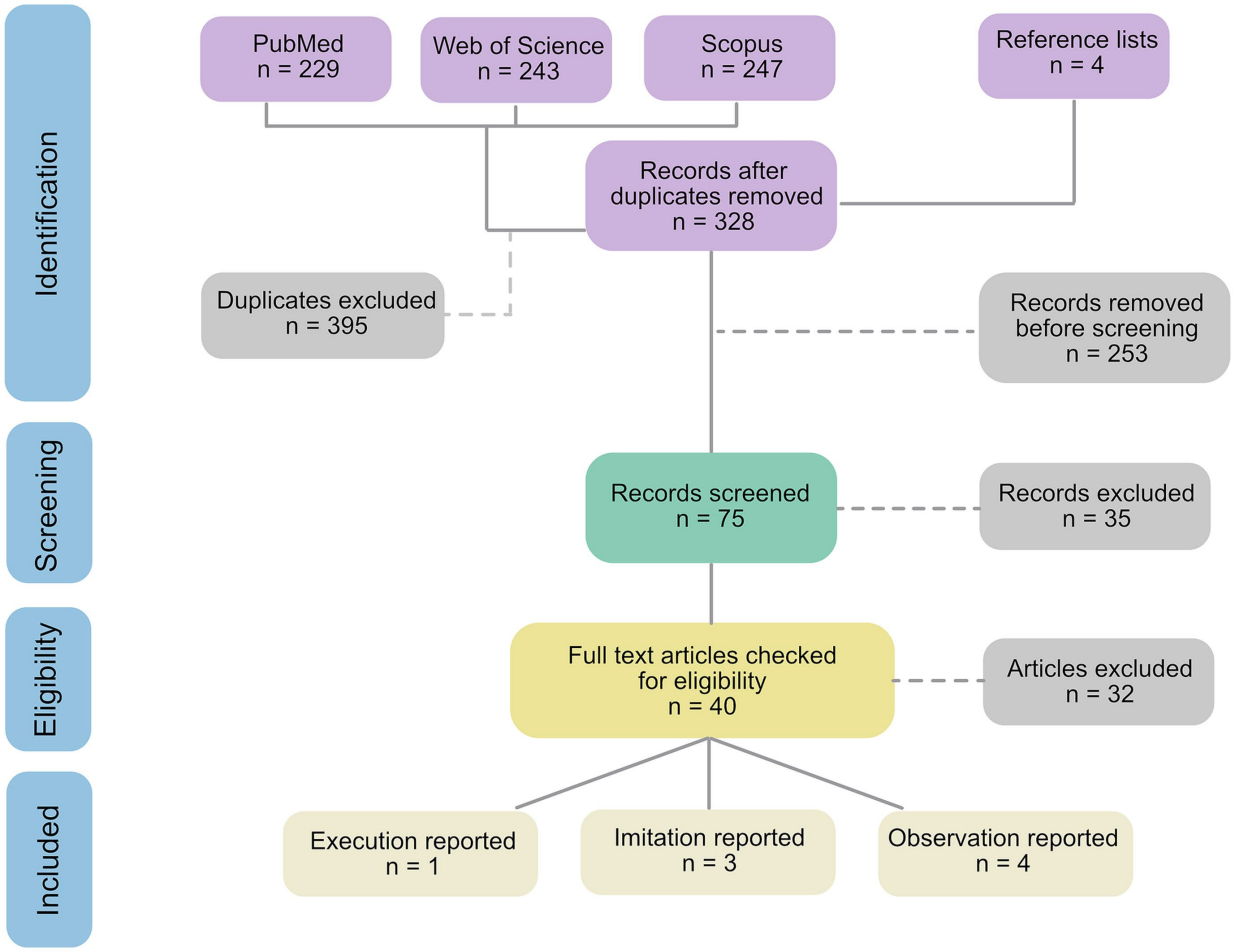
PRISMA flow diagram for the systematic review.

The search resulted in 328 studies after removing duplicates. Studies were evaluated and selected as illustrated in Figure 1, with eight studies that in the end met criteria for inclusion. One additional fMRI-study (Kilroy et al., 2021) involving observation, imitation, execution and mentalizing tasks in autistic children appeared to meet the inclusion criteria but eventually had to be excluded. This was because the experimental condition of interest for this review (i.e., hand actions) was not analyzed on its own but collapsed with the other experimental conditions (emotional faces, non-emotional faces) for each task.

### Study characteristics

The included studies were published between 2006 and 2023, all in different scientific journals and summarized in Table 1. One study (Mostofsky et al., 2009) focused solely on action execution; three (Jack and Morris, 2014; Wadsworth et al., 2017; Williams et al., 2006) on imitation; and four (Fourie et al., 2020; Knaus et al., 2023; Pokorny et al., 2015, 2018) on observation. Two of the studies focusing on imitation (Jack and Morris, 2014; Wadsworth et al., 2017; Williams et al., 2006) also included execution and observation in their experimental paradigms. Two studies (Mostofsky et al., 2009; Williams et al., 2006) used 1.5 T scanners whereas the others used 3 T scanners.

**Table 1:** Overview of included studies.

**Study**	**Overview of experiment**	**Scanning and preprocessing**	**Behavioral findings**	**Significant between-group differences**
Studies focusing on action execution				
Mostofsky et al. (2009)	Aim To examine neural function and connectivity during self-paced sequential finger-tapping in children with ASD Design Blocked Participants tapped each finger to the thumb in 30 s-long blocks of self-paced finger sequencing with the right or left hand and equally long resting blocks in between	Scanner 1.5 T, Philips Spatial template Study specific template based on grey-matter and functional images from all participants Voxel size 2 mm^3^	No significant group differences in time to complete five sequences of right hand or left hand finger tapping outside the scanner No significant difference in frequency of right hand taps in the scanner, however significantly lower frequency of left hand taps for the ASD group	Motor task > Rest TD > ASD: Ipsilateral anterior cerebellum for RH and LH execution; right posterior/inferior cerebellum, and left lingual/fusiform gyrus for RH execution
Studies focusing on imitation				
Jack and Morris (2014)	Aim To examine if interactions between cerebellar Crus I and posterior superior temporal sulcus predicted weaker mentalizing ability in adolescents with ASD Design Event-related Participants either passively viewed a model performing a random four finger sequence, imitated the sequence, or performed a sequence as indicated by visuospatial cues	Scanner 3 T, Siemens Trio Spatial template MNI Voxel size 3 mm x 3 mm x 4.2 mm	No significant differences between groups in fMRI task performance in terms of response latency and error rate	Imitation > Baseline TD > ASD: Left cerebellar VI, right Crus I Imitation > Execution TD > ASD: Right occipital pole, right superior lateral occipital cortex, left inferior lateral occipital cortex, right IFG (pars opercularis)
Wadsworth et al. (2017)	Aim To examine the role of the ‘action imitation network’ as a mediator for motor imitation in children and adolescents with ASD Design Event-related Participants used their right hand to imitate hand gestures displayed as still pictures, or relaxed their hand while viewing a cross (baseline)	Scanner 3 T, Siemens Allegra Spatial template MNI Voxel size 3 mm x 3 mm x 3 mm	No differences between groups in post-scan test of imitation ability	Imitation > Rest TD > ASD: Left middle cingulate, right precentral gyrus, right angular gyrus
Williams et al. (2006)	Aim To test the hypothesis that neural systems involved in imitation, particularly the mirror-neuron system, function atypically in individuals with ASD Design Blocked Participants raised index or middle finger in execution and imitation conditions, or simply viewed visual stimuli in observation conditions	Scanner 1.5 T, GE Medical Systems Spatial template A non-specified standard template Voxel size 2 mm x 2 mm x 2 mm	Task performance was close to 100% for all participants	Imitation > Rest TD > ASD: Right fusiform gyrus, right middle occipital gyrus, right IPL, right lingual gyrus, right middle temporal gyrus, left IPL ASD > TD: Bilateral superior temporal gyrus, right parahippocampal gyrus, right cingulate gyrus, left uncus, left precentral gyrus, left claustrum, left middle frontal gyrus, left middle occipital gyrus Imitation > Execution TD > ASD: left IPL, left anterior cingulate, right precuneus ASD > TD: left middle frontal gyrus, left precentral gyrus, left sub gyral region, left superior parietal lobule, right middle frontal gyrus, right precentral gyrus
Studies focusing on observation				
Fourie et al. (2020)	Aim Investigations of brain activity during gesture processing, gesturing ability and social symptomology in ASD compared with TD Design Blocked Participants observed a 3D human avatar performing different gestures, and then selected one out of two presented words that best described the performed action	Scanner 3 T, Siemens Trio Spatial template MNI 152 Voxel size 3.4 mm^3^	No group differences in response times or accuracy for recognition of gestures	No significant group differences
Knaus et al. (2023)	Aim To investigate AON activity in ASD compared with TD in an action-viewing task, and if prevalence of potential deviances in AON in ASD was associated with language deficits Design Blocked Participants watched video clips of a person (arm and hand only) performing actions on objects, or viewed a crosshair (baseline)	Scanner 3 T, Siemens Verio Spatial template MNI 152 Voxel size Not reported	No group differences in accuracy for verbal-gesture integration or post-scan accuracy of the fMRI task	No significant group differences
Pokorny et al. (2015)	Aim Investigations of AON activity in ASD compared with TD in object directed and non-object directed actions Design Blocked Participants watched video clips of a person (arm and hand only) performing reach and grasp actions either on objects or without objects, with the end of the reach/grasp either visible or occluded	Scanner 3 T, Siemens Trio Spatial template MNI Voxel size 3.4 mm^3^	No behavioral data collected during experiment or post scanning	No significant group differences
Pokorny et al. (2018)	Aim Investigations of AON and MZN activity in ASD compared with TD while viewing actions Design Slow event-related Participants watched video clips of a female actor performing eating and placing actions in either a conventional or unconventional way	Scanner 3 T, Siemens Trio Spatial template MNI Voxel size 3.4 mm^3^	No behavioral data collected during experiment or post scanning	Conventional eating > baseline TD > ASD: right temporoparietal junction, left temporoparietal junction, posterior cingulate cortex and dorsomedial prefrontal cortex

T = Tesla; MNI = Montreal Neurological Institute.

### Risk of bias in studies

Overall, the included studies showed relatively small differences in quality assessment scores (range: 21–25). In terms of participant descriptions, all studies provided adequate information regarding sample characteristics. All studies recruited participants with an autism diagnosis, and diagnostic verification was conducted for all ASD participants using standardized assessment tools. In the studies by Pokorny et al. (2015, 2018) a limited number of participants did not have their diagnosis verified. Four studies (Fourie et al., 2020; Knaus et al., 2023; Pokorny et al., 2015, 2018) did not report co-occurring clinical conditions or medication status in detail. In these studies, inclusion criteria were nevertheless restricted by excluding participants with neurological or medical conditions (Fourie et al., 2020), use of antipsychotic medication (Fourie et al., 2020; Pokorny et al., 2015, 2018), neurological damage, known genetic disorders, or a history of seizures or prematurity (Knaus et al., 2023; Pokorny et al., 2015, 2018). Reporting on sampling procedures was limited. None of the studies provided sufficiently detailed information about sampling methods, and only one study (Mostofsky et al., 2009) described procedures that were judged unlikely to introduce selection bias. For five studies (Fourie et al., 2020; Knaus et al., 2023; Mostofsky et al., 2009; Wadsworth et al., 2017; Williams et al., 2006) recruitment of TD participants appeared to originate from similar contexts as the ASD groups, whereas three studies (Jack and Morris, 2014; Pokorny et al., 2015, 2018) did not adequately describe this process. All studies were comparable in terms of demographic variables (age, sex) and at least one IQ measure; where group differences in IQ existed, IQ was included as a covariate in the fMRI analyses. Methodologically, all studies met basic criteria for imaging quality (≥1.5 T magnet strength, adequate sample sizes, clearly described acquisition and preprocessing pipelines, reporting in standardized coordinate spaces, and transparent statistical procedures), details are provided in Supplementary Table S2. Nevertheless, inconsistencies were observed across studies regarding: reporting of dropouts, if whole brain analyzes were automated and performed without a-priori regional selections, slice thickness, cluster-forming thresholds, application of multiple comparison corrections, clarity of baseline conditions, clarity in contrast definitions, and whether conclusions were fully supported by reported results.

Based on our quality assessment findings, the overall risk of bias across studies is low, but variation in reporting practices and methodological parameters should be taken into consideration in the synthesis of results. Detailed quality assessment outcomes for each study are provided in the Supplementary Table S1.

### Participant characteristics

#### Age and sex distributions

In total, selected studies included 129 ASD participants and 128 TD participants. Participant characteristics are presented in Table 2. Group mean ages spanned between 10.5–15.5 years and all studies but one reported age ranges where all participants were < 18 years of age. One study did not include the age range of their participants, but report the group mean ages 15.4 years (ASD) and 15.5 years (TD) and refer to their participants as “adolescent or young adult men.” With the exception for one study that only included male participants (Williams et al., 2006), all studies had mixed-sex groups with a majority of boys. One study (Fourie et al., 2020) reported recruiting 4/3 girls and 15/17 boys for the ASD and TD groups, respectively. However, the final number of girls and boys was not specified after a total of six participants (sex not specified) were excluded from fMRI analyses. The other six studies with mixed-sex groups (Mostofsky et al., 2009; Jack and Morris, 2014; Wadsworth et al., 2017; Knaus et al., 2023; Pokorny et al., 2018, 2015) included 15 (ASD)/21(TD) girls, and 83 (ASD)/76 (TD) boys. Sample sizes ranged from 13 ASD + 13 TD participants (Mostofsky et al., 2009) to 23 ASD + 20 TD participants (Knaus et al., 2023).

**Table 2:** Participant characteristics.

**Study**	Group	***N* (M/F)**	**Confirmation of diagnosis**	**Age range;** ***M* age (SD)**	**IQ *M (SD)***	**Handedness**	**Behavioral assessments *M* (SD)**
Mostofsky et al. (2009)	ASD	13 (11/2)	ADI-R ADOS-G	8–12; 10.9 (1.5)	FSIQ: 103 (108)* N-VIQ: 106 (18)	100% RH	PANESS: 40.1 (12.8)^2^*
	TD	13 (11/2)	N/A	8–1 2; 10.5 (1.4)	FSIQ: 118 (14)* N-VIQ: 120 (19)	100% RH	PANESS: 18.8 (8.7)*
Jack and Morris (2014)	ASD	15 (13/2)	ADOS	12–17; 14.2 (1.6)	FSIQ:110.5 (15) VIQ: 109.3 (17.3) PIQ: 110.3 (14.9)	EHI: 57.1 (62.4)	SCQ: 23.7 (5.9)* SRS Total: 83.5 (12.5)*
	TD	15 (11/4)	N/A	12–17; 13.8 (1.7)	FSIQ: 112.3 (8) VIQ: 114.3 (10.2) PIQ: 107.6 (10.5)	EHI: 50.2 (48.3)	SCQ: 1.8 (2)* SRS Total: 42.6 (5.9)*
Wadsworth et al. (2017)	ASD	14 (12/2)	ADI ADOS	8–17; 12 (2.9)	FSIQ: 110 (16.7) VIQ: 109 (17.6) PIQ: 108 (15.1)	100% RH	AQ: 72 (33)* VMI: 95 (10.3) PANESS: 53 (9.9)
	TD	15 (11/4)	N/A	9–15; 12 (1.6)	FSIQ:102 (16.2) VIQ: 98 (17.6) PIQ: 99 (9.6)	100% RH	AQ: 35 (27.3)* VMI: 95 (9.7) PANESS: 50 (12)
Williams et al. (2006)	ASD	16 (16/0)	ADI-R ADOS-G	N/A; 15.4 (2.2)	FSIQ:100.4 (21.7) VIQ: 100.9 (23.4) PIQ: 98.4 (19.0)	100% RH EHI: 74.7 (20.0)*	N/A
	TD	15 (15/0)	N/A	N/A; 15.5 (1.6)	FSIQ: 99.7 (18.3) VIQ: 101.3 (21.1) PIQ: 97.6 (13.9)	100% RH EHI: 93.1 (7.5)*	N/A
Fourie et al. (2020)	ASD	15 (N/A)^1^	ADOS	10.5–15.8; 13.5 (1.7)	PIQ: 107.1 (14.1) VIQ: 106.8 (13.2)	80% RH	SRS: 76.7 (15.3)* SCQ: 22 (7.1)* DCDQ: 41.6 (12.4)*
	TD	16 (N/A)^1^	N/A	9.6–16.9 ;12.7 (2.3)	PIQ: 110.3 (12.0) VIQ: 115.1 (16.0)	93.8% RH	SRS: 44.2 (7.8)* SCQ: 2.2 (2.8)* DCDQ: 70.5 (7.1)*
Knaus et al. (2023)	ASD	23 (19/4)	ADOS-2 ADI-R or SCQ	11–17; 13.8 (1.9)	VIQ: 90.1 (12.6)* N-VIQ: 100.8 (13.3)*	87% RH AHA: 2. (1.6)	N/A
	TD	20 (17/3)	N/A	11–17; 12.8 (1.6)	VIQ: 105.8 (10.6)* N-VIQ: 110.4 (10.9)*	85% RH AHA: 2.3 (1.1)	N/A
Pokorny et al. (2015)	ASD	17 (14/3)	ADOS	10.3–17.6; 14.6 (2.2)	PIQ: 105.5 (14.1)	N/A	SCQ: 23.73 (7.23)* DCDQ: 46.3 (13.7)*
	TD	18 (14/4)	N/A	9.1–17.7; 14.3 (2.2)	PIQ: 111.9 (12.9)	N/A	SCQ: 1.7 (2.2)* DCDQ: 69.3 (7.5)*
Pokorny et al. (2018)	ASD	16 (14/2)	ADOS	11.6–17.6; 14.9 (2)	PIQ: 105.5 (12.6)^2^	N/A	SCQ: 23 (6.8)^2^* DCDQ: 45.3 (14)^2^*
	TD	16 (12/4)	N/A	9.2–17.7; 14.2 (2.3)	PIQ: 110.8 (12.6)^2^	N/A	SCQ: 1.87 (2.3)^2^* DCDQ: 69.1 (7.8)*

*indicates a statistically significant difference between groups (p < 0.05). Footnotes: ^1^ Sex not specified for participants included in fMRI analyses; ^2^ Data missing from 1–3 participants. Abbreviations: ASD, Autism spectrum disorder; TD, Typically developing; N, Number; M/F, Male/Female; M, Mean; SD, Standard deviation; IQ, Intelligence Quotient (P, Performance; V, Verbal; N-V, Non-verbal; FS, Full-scale); RH, Right-handed; EHI, Edinburgh Handedness Inventory; AHA, Almli Handedness Assessment; N/A, Not available (data not presented or test not administered); SRS, Social Responsivity scale; SCQ, Social Communication Questionnaire; ADOS, Autism Diagnostic Observation Schedule (2, Second edition; G, Generic); ADI-R, Autism Diagnostic Interview-Revised; AQ, Autism Spectrum Quotient; DCDQ, Developmental Coordination Disorder Questionnaire; PANESS, The Physical and Neurological Examination for Soft Signs; VMI, Visual-Motor Integration.

#### ASD diagnosis

All studies included participants with pre-established ASD diagnosis, recruited from local communities, clinics, special schools, or similar (Fourie et al., 2020; Jack and Morris, 2014; Knaus et al., 2023; Mostofsky et al., 2009; Pokorny et al., 2015, 2018; Wadsworth et al., 2017; Williams et al., 2006). All studies reaffirmed ASD diagnosis using clinically approved approaches and assessments; specifics of these can be found in Table 2. All control groups consisted of non-autistic participants. Five studies also specified autism in the immediate family as an exclusion criterion for TD participants, or stated that none of the TD participants had history of developmental delay or immediate family members diagnosed with autism (Knaus et al., 2023; Mostofsky et al., 2009; Pokorny et al., 2015, 2018).

#### Intelligence quotient

All studies presented either full-scale IQ (FSIQ) (Jack and Morris, 2014; Mostofsky et al., 2009; Wadsworth et al., 2017; Williams et al., 2006) or selected aspects of it, i.e., performance IQ (PIQ) (Jack and Morris, 2014; Wadsworth et al., 2017; Williams et al., 2006; Fourie et al., 2020; Pokorny et al., 2015, 2018) visual IQ (VIQ) (Fourie et al., 2020; Knaus et al., 2023), non-visual IQ (N-VIQ) (Knaus et al., 2023) measured using standardized test batteries. All studies included participants with an assessed IQ of 70 or above on selected IQ indices or on FSIQ, see Table 2.

#### ASD traits

Five studies included self- or parent ratings of autism traits as participant descriptives, specifically: the Social Responsivity Scale (SRS) (Fourie et al., 2020; Jack and Morris, 2014), the Social Communication Questionnaire (SCQ) (Jack and Morris, 2014; Fourie et al., 2020; Pokorny et al., 2015, 2018) the Autism-spectrum Quotient (AQ) (Wadsworth et al., 2017), and the Theory of Mind Inventory (ToMI) (Jack and Morris, 2014). All comparisons showed significant group differences, i.e., the ASD groups had more difficulties in social communication and increased levels of autism-related behaviors; scores are presented in Table 2.

#### Motor abilities

Assessments and group comparisons of motor abilities were included as descriptive measures in some of the studies, presented in Table 2. Three studies used the Developmental Coordination Disorder Questionnaire (DCDQ) and reported significantly lower motor abilities in the ASD groups (Fourie et al., 2020; Pokorny et al., 2015, 2018); two studies included the Physical and Neurological Examination for Soft Signs (PANESS), where one study found no group differences (Wadsworth et al., 2017) and one study reported significantly lower motor abilities in the ASD group (Mostofsky et al., 2009); one study used the Visual-Motor Integration test (VMI) and found no significant difference between groups (Wadsworth et al., 2017).

#### Handedness across studies

Three studies included only right-handed participants (Mostofsky et al., 2009; Wadsworth et al., 2017; Williams et al., 2006), one of which reported handedness quotient scores which were significantly lower (i.e., less right-dominant) in the ASD than TD group (Williams et al., 2006); two studies included both right- and left-handed participants (Fourie et al., 2020; Knaus et al., 2023), of which one also presented similar laterality quotients between groups (Knaus et al., 2023); one study did not enclose number of right/left-handed participants but included laterality quotients that did not differ between groups (Jack and Morris, 2014); two studies did not report handedness or laterality quotients (Pokorny et al., 2015, 2018). Handedness distribution and laterality quotient scores are presented in Table 2.

### Results of studies focusing on action execution

#### Paradigms and procedures

Mostofsky et al. (2009) was the only included study that focused exclusively on movement execution. This study employed a finger sequencing paradigm where participants moved their fingers (pressing one finger at a time against the thumb) in a fixed sequential order, prompted by text presented on a screen. The task utilized a blocked design, consisting of 30-s-long blocks of right-hand execution, left-hand execution, and a resting baseline. The three block types were performed in a specific order (counterbalanced between subjects) for a total of four cycles. Contrasts of interest were right-hand execution vs. rest, and left-hand execution vs. rest, in TD vs. ASD. Functional connectivity was also investigated, analyzed by time-course correlations of activation in relevant regions of interest (ROI) that together constituted motor circuits related to execution of the finger-tapping task.

#### Behavior

Group comparisons of the number of in-scanner finger sequencing taps (tapping rate), showed no group difference in tapping rate for right-handed tapping but significantly higher left-handed tapping rate in the TD group (Mostofsky et al., 2009). Since there were also significant group differences in FSIQ (TD > ASD), tapping rate and FSIQ were used as covariates in additional analyses of ROI activation.

#### Brain activity

##### Execution vs. resting baseline

**Figure 2:**
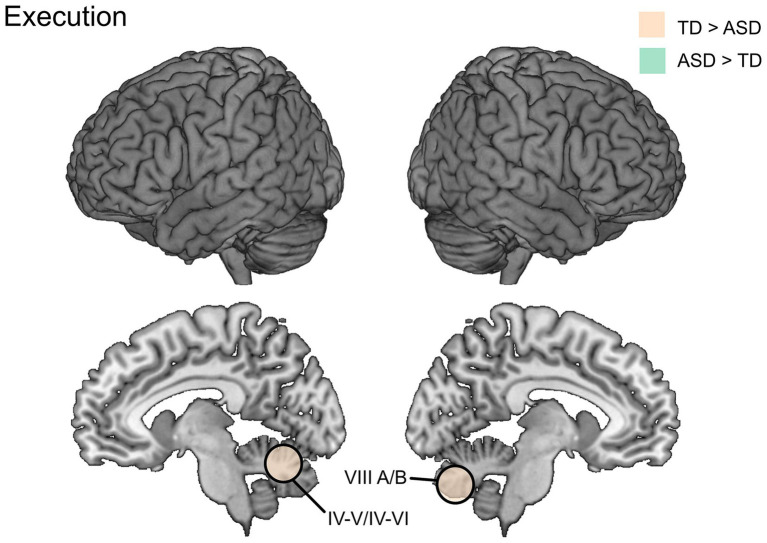
Differences in brain activation between ASD and TD groups during execution vs. baseline as reported by Mostofsky et al. (2009).

Separate sets of ROIs were created for the right-hand execution vs. rest (*n* = 10) and left-hand execution vs. rest (n = 8) contrasts, based on overlapping activity between TD and ASD single-group maps from whole-brain analyses (Mostofsky et al., 2009). Group comparisons of the ROI-activations showed that the TD group had higher activation than ASD in the right posterior/inferior cerebellum (lobule VII A/B), and the left anterior cerebellum (lobules IV/V) in the right vs. rest analysis. The left vs. rest contrast showed higher activation for TD than ASD in the left anterior cerebellum (lobules IV/VI). Results are presented in Table 3 and Figure 2.

**Table 3:** Between-groups differences for execution, imitation and observation conditions compared to baseline.

Study domain	Author and year	Group difference	Region	*t*-value	*Z*-score	*k*	x	y	z	BA	Hem
Execution	Mostofsky et al. (2009)	TD > ASD	Posterior inferior cerebellum (Lobule VIII A/B)	3.9	*NR*	*NR*	*NR*	*NR*	*NR*	-	Right
			Anterior cerebellum (Lobules IV/V)	3.39	*NR*	*NR*	*NR*	*NR*	*NR*	-	Left
			Anterior cerebellum (Lobules IV/VI) *(Left hand execution)*	3.33	*NR*	*NR*	*NR*	*NR*	*NR*	-	Left
Imitation	Jack and Morris (2014)	TD > ASD	Cerebellar VI	*NR*	3.97	702	−20	−64	−32	-	Left
			Crus I	*NR*	3.89	547	42	−58	−28	-	Right
	Wadsworth et al. (2017) ^1^	TD > ASD	Middle cingulate	4.7	*NR*	89	−4	−8	34	24	Left
			Angular gyrus	4.8	*NR*	76	48	−50	30	39	Right
			Precentral gyrus	3.7	*NR*	36	36	4	30	44	Right
			Middle cingulate *(Mask)*	3.7	*NR*	41	−6	−6	40	24	Left
			Precentral gyrus *(Mask)*	3.7	*NR*	32	36	4	30	44	Right
	Williams et al. (2006) ^2^	TD > ASD	Fusiform gyrus	4.96	4.21	326	38	−51	−11	37	Right
			Middle occipital gyrus	3.86	3.45	206	24	−84	−4	18	Right
			Inferior parietal lobule	3.22	2.96	16	46	−36	59	40	Right
			Lingual gyrus	3.09	2.86	29	28	−66	−2	19	Right
			Middle temporal gyrus	2.79	2.61	24	50	−62	9	37	Right
			Inferior parietal lobule	3.64	3.28	48	−53	−56	40	40	Left
		ASD > TD	Superior temporal gyrus	3.77	3.38	100	44	3	−15	38	Right
			Parahippocampal gyrus	2.81	2.62	20	20	−18	−18	28	Right
			Cingulate gyrus	2.79	2.61	12	20	26	21	32	Right
			Uncus- amygdala	4.37	3.81	37	−20	−1	−22	36/Amyg	Left
			Superior temporal gyrus	3.98	3.53	70	−40	−1	−10	21	Left
			Precentral gyrus	3.33	3.04	57	−20	−22	64	4	Left
			Claustrum	2.95	2.75	35	−28	7	16	-	Left
			Middle frontal gyrus	2.93	2.73	61	−30	8	53	6	Left
			Middle occipital gyrus	2.71	2.54	10	−44	−77	8	19	Left
Observation	Pokorny et al. (2018) ^1^	TD > ASD	Temporoparietal junction	4.83	4.12	265	44	−56	26	39	Right
			Temporoparietal junction	3.36	3.07	108	−52	−66	28	39	Left
			Posterior cingulate cortex	4.5	3.9	214	4	−56	36	31	Right
			Dorsomedial prefrontal cortex	3.76	3.38	70	4	50	16	10	Right

k = cluster size; x,y,z = coordinates in MNI-space unless otherwise indicated; BA = Brodmann areas; Hem = hemisphere; NR = Not Reported; Amyg = Amygdala; ^1^ BA estimated from coordinates by authors using BioImage Suite Web (Papademetris et al., 2006); ^2^ Talairach coordinates reported.

##### Associations between behavioral measures and brain activity

Analyses with added covariates were performed to control for tapping rate (alone) and FSIQ (together with tapping rate), revealing an additional significant group difference for the right-hand execution vs. rest contrast (TD > ASD) in the left lingual/fusiform gyrus (BA18/9) with tapping rate as a covariate (Mostofsky et al., 2009). However, this difference was not statistically significant when also adding FSIQ as covariate.

##### Functional connectivity

Seven ROIs constituting motor circuits of connectivity were localized to the bilateral primary motor cortex (M1), bilateral anterior cerebellum, bilateral thalamus, and supplementary motor area (SMA) (Mostofsky et al., 2009). Control ROIs located in the right auditory cortex and brainstem were also analyzed to verify the specificity of motor circuit connectivity. Both groups showed differences in connectivity between motor- and control circuits, and there were no group differences in control circuit connectivity. A time-course analysis of the whole scanning session revealed significantly higher connectivity in the motor circuits for the TD than the ASD group. Separate analyses of conditions (right-hand execution; left-hand execution; rest) showed robust differences in connectivity during both right- and left-hand execution, but only marginally significant differences during rest. When comparing each ROI individually against each of the other ROI’s (time-course correlations), a main-effect of group (TD > ASD) was evident in all ROI-pairs except for right and left cerebellum. Furthermore, significant interaction between group and condition was seen in the left and right thalamus pair, left thalamus and SMA, and right thalamus and SMA, where the ASD group showed higher connectivity in those region pairs during rest than during right- and left-handed execution.

### Results of studies focusing on imitation

#### Paradigms and procedures

Three studies focused on imitation (Jack and Morris, 2014; Wadsworth et al., 2017; Williams et al., 2006). All studies had an imitation vs. baseline contrast, of which two also included contrasts where imitation was compared to execution and/or observation conditions (Jack and Morris, 2014; Williams et al., 2006).

Jack and Morris (2014) investigated imitation in relation to observation and execution in a slow event-related design. For imitation and observation trials, visual stimuli consisted of videos showing a left hand from a third-person view performing a randomized sequence of finger presses on a keypad, while participants were instructed to either imitate the action on a button box (imitation) or to passively view the video without acting (observation). In execution trials, an additional white circle on the presented keypad indicated the button sequence to be executed. The authors were primarily interested in the non-motor aspect of imitation, thus the imitation > execution contrast. The paradigm also included an unspecified baseline condition.

Wadsworth et al. (2017) focused exclusively on imitation vs. a crosshair baseline condition in a blocked design. Participants were shown still images of hand gestures, presented in first-person view, and were instructed to replicate the gestures using their right hand. Imitation < baseline analyzes were carried out to compare whole-brain activation and in a mask made of *a priori* ROIs that together constitute an ‘action imitation network’. These ROIs were located in bilateral IFG, bilateral posterior middle temporal gyrus, left dorsal premotor cortex (PMC), left M1, bilateral somatosensory cortex, bilateral occipital cortex, right inferior parietal lobule (IPL), right insula, and right inferior temporal gyrus.

Williams et al. (2006) used a blocked design with a resting baseline and observation and execution (including imitation) conditions, using the same three stimulus types for both conditions: animation (moving gray-scale pictures of a hand in third-person view raising the index- or middle finger), symbolic cue (a still image of the same hand with a cross on the fingernail of the index- or middle finger), and spatial cue (a gray background with a cross on either the right or the left side of the screen). Participants were asked to observe the stimuli without acting (observation condition), or to lift the index- or middle finger as indicated by the animation (imitation condition) or by the symbolic/spatial cue (action execution condition: the average activation from symbolic and spatial cues).

#### Behavior

All three imitation studies assessed task performance/adherence, either by analyses of in-scanner performance measured as response latency and error rate (Jack and Morris, 2014), video recordings of performance (Williams et al., 2006), or by a post-scanning assessment of the ability to imitate hand gestures (Wadsworth et al., 2017). All assessments showed high adherence to tasks and no significant differences in performance between groups (Jack and Morris, 2014; Wadsworth et al., 2017; Williams et al., 2006).

#### Brain activity

##### Imitation vs. baseline

Whole-brain analyses contrasting imitation > baseline were conducted in all three studies, using Z statistics (Jack and Morris, 2014) or two sample *t*-tests (Wadsworth et al., 2017; Williams et al., 2006). Jack and Morris (2014) found group differences (TD > ASD) in two cerebellar regions; left cerebellar VI (with local peaks in vermis VIIIa, vermis IX, and cerebellar V), and right crus I (with local peaks in VIIb and Crus II). Both Williams et al. (2006) and Wadsworth et al. (2017) reported significantly higher activation for TD than ASD groups in regions of the IPL. Specifically, Williams et al. (2006) observed this effect in the right supramarginal gyrus (BA40) and the left-hemisphere border between angular and supramarginal gyri (BA40), while Wadsworth et al. (2017) identified a similar pattern in the right angular gyrus (BA39). Williams et al. (2006) further found significant differences (TD > ASD) in the right fusiform (BA37) and lingual (BA19) gyri, right secondary visual cortex (BA18), whereas Wadsworth et al. (2017) also reported differences (TD > ASD) in the left middle cingulate (BA24) and in the right precentral gyrus/pars opercularis (BA44).

Higher activation for the ASD than TD group in the imitation > baseline contrast was reported by Williams et al. (2006) in the right (BA38) and left (BA21) superior temporal gyrus, right parahippocampal gyrus (BA28), right cingulate gyrus (BA32), left uncus/amygdala, left M1 (BA4) and PMC (BA6) areas, left claustrum, and left middle occipital gyrus (BA19).

**Figure 3:**
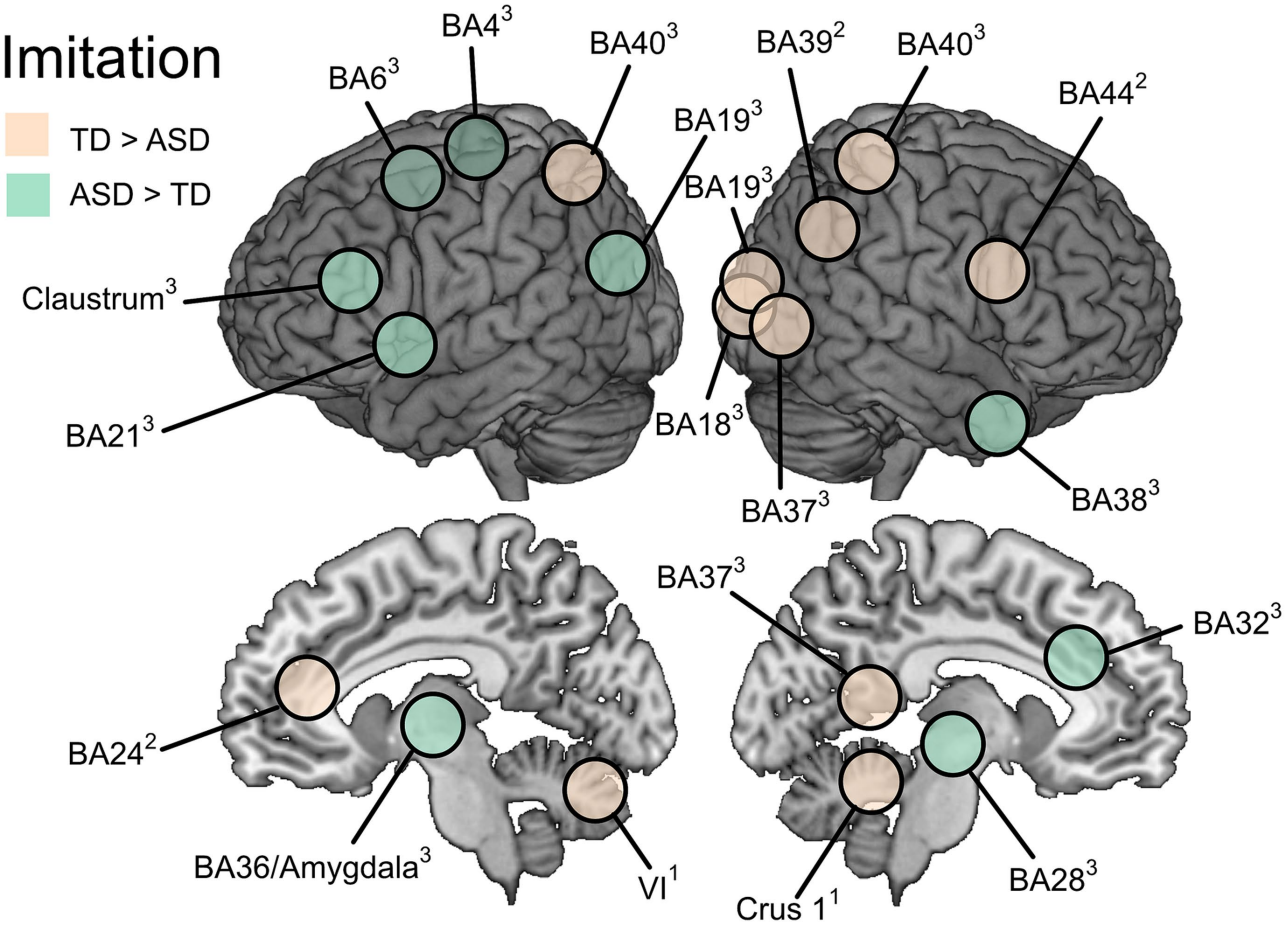
Differences in brain activation between ASD and TD groups during imitation vs. baseline. Superscript numbers denote the publication reporting the result: Jack and Morris (2014)^1^, Wadsworth et al. (2017)^2^, and Williams et al. (2006)^3^.

The masked analysis of the imitation > baseline contrast (Wadsworth et al., 2017) showed increased activation for TD compared to ASD in two of the three regions that differed between groups in the whole-brain analysis: the left middle cingulate (BA24) and the right pars opercularis (BA44) (Figure 3).

##### Imitation vs. execution

To single out non-motor aspects of imitation, two studies analyzed imitation > execution contrasts, where Jack and Morris (2014) analyzed whole-brain activation whereas Williams et al. (2006) performed a masked analysis limited to frontal and parietal regions and further in specific ROIs. Jack and Morris (2014) showed higher activation for TD compared to ASD in four relatively large clusters (*k* ranges between 612 and 319). Local activation peaks occurred in the right occipital pole, right superior lateral occipital cortex, left inferior lateral occipital cortex, and in a right hemisphere region in the right IFG (pars opercularis). Analysis of task-related connectivity showed no significant group effects (Jack and Morris, 2014).

In the masked analysis where Williams et al. (2006) compared activation in frontal and parietal regions (imitation > execution), TD had significantly higher activation than ASD in the left IPL, and smaller clusters in the left anterior cingulate cortex and right precuneus. The reversed contrast (ASD > TD) revealed seven clusters of significantly higher activation in the ASD than TD group, out of which the three largest clusters were located in left PMC areas. There was also another smaller cluster of activation in the left precentral gyrus, one in the left superior parietal lobule, and two clusters around the right middle frontal / precentral gyri.

##### Imitation vs. observation

To disentangle imitation effects from those related to visual processing, Williams et al. (2006) performed masked between-group analyses confined to occipital and temporal lobes in the contrast of imitation > observation. In these analyses, TD had higher activity than ASD bilaterally in the lingual gyrus and bilateral middle temporal gyrus. The reversed contrast (ASD > TD) did not show any effects. Follow up ROI analyses on the imitation > observation contrast, focusing on the right middle-temporal gyrus region, showed activation during imitation but not observation in the TD group, whereas the ASD group showed the reversed pattern.

##### Associations between behavioral measures and brain activity

One study (Wadsworth et al., 2017) examined relations between whole-sample brain activation and the two behavioral measures PANESS (motor skills) and AQ (ASD symptom severity). They found that AQ scores were significantly negatively related to parameter estimates in the right IPL.

### Results of studies focusing on observation

#### Paradigms and procedures

A total of four studies focused on brain activity related to observing goal-directed actions (Fourie et al., 2020; Knaus et al., 2023; Pokorny et al., 2015, 2018). The two studies by Pokorny et al. (2015, 2018) both employed event-related designs. In the first of these (Pokorny et al., 2015), participants observed videos of goal-directed actions performed by an actor reaching in from the right or the left (only hand and arm showing). Actions were directed either toward an object (transitive) or towards a location without an object present (intransitive). Furthermore, the end-state of the performed task was occluded in half of the trials, thus resulting in four conditions (visible/occluded + transitive/intransitive). Focus was on group differences (ASD vs. TD) in activity in specific ROIs located in the AON, in relation to observing transitive vs. intransitive actions. The study by Pokorny et al. (2018) used video stimuli where an actor performed placing and eating actions in conventional or unconventional ways, for example eating a green apple (conventional) or a tennis ball (unconventional); placing a tennis ball in a tennis ball container (conventional) or a green apple in the same (unconventional). The actor was seated by a table and filmed from the front with the whole upper body and head visible. Between-group comparisons of brain activity were performed on the contrasts conventional eating > baseline, and conventional placing > baseline, in three masked analyses in the areas: AON, mentalizing network (MZN), and superior temporal sulcus (STS).

Fourie et al. (2020) had a blocked design where participants observed video clips of a human-like avatar performing 11 different actions. These were based on motion capture of a human actor (i.e., naturalistic, human actions performed by a digital avatar) and presented in full body from a third-person perspective. Actions were classified as functional pantomimes (*N* = 5, e.g., driving) or communicative gestures (*N* = 6, e.g., waving), and performed at three intensity levels (low, medium, high). After each 5 s clip, participants were instructed to choose which descriptive word (out of two presented) that most closely matched the performed action. Analyses compared each of the six conditions (2 movement types x 3 movement intensity levels) with baseline, and focused on whole-brain comparisons, and comparisons of three *a priori* ROIs: lateral occipital temporal cortex, posterior superior temporal sulcus, and the AON.

In the study by Knaus et al. (2023), participants passively watched video clips showing an actor (only arms and hands visible, presented in third-person view) performing actions on objects, for example playing with a toy car. Stimuli blocks consisted of six action clips for a total of 21 s, and baseline blocks consisted of equally long crosshair rest. One aim of the study was to examine activations in the posterior IFG (including ventral PMC, POP; and rostral inferior parietal lobule, SMG) during action observation in ASD vs. TD adolescents. A whole-brain comparison of activation between groups was also performed, and non-verbal IQ was used as a covariate-of-no-interest in all analyses. Another aim of the study by Knaus et al. (2023) was to investigate if impairments in the AON were associated with language deficits in ASD. This was investigated by ASD subgroup (low language ability vs. high language ability) direct group comparisons, not further reported here since these analyses are outside the scope of this review.

#### Behavior

Accuracy in the fMRI task was presented in two studies (Fourie et al., 2020; Knaus et al., 2023) showing no group differences. Similarly, the ability to produce the observed gestures outside of the scanner (Fourie et al., 2020) and performing a verbal-gesture integration task (Knaus et al., 2023) showed no group differences. However, communicative gestures yielded significantly more incorrect responses and significantly longer response times than functional gestures for both groups (Fourie et al., 2020).

#### Brain activity

##### Observation vs. baseline

**Figure 4:**
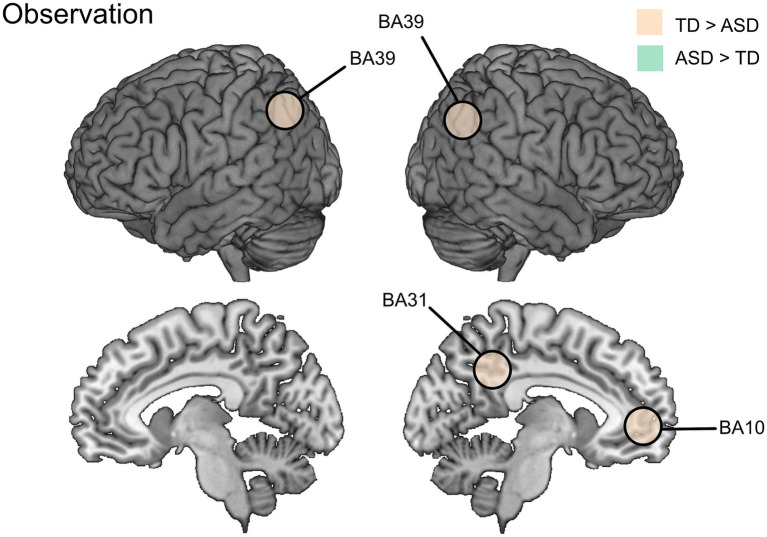
Differences in brain activation between ASD and TD groups during observation vs. baseline as reported by Pokorny et al. (2018).

In Pokorny et al. (2015) group comparisons (TD vs. ASD) on the contrast between the undefined baseline and each observational condition (transitive/intransitive + visible/occluded) revealed no significant group effects in the specified ROIs within the AON. Similarly, there were no significant group differences in the conventional placing > baseline contrast (Pokorny et al., 2018), but the conventional eating > baseline contrast yielded significant group differences (TD > ASD) in ROIs located in the MZN: activity was higher for the TD than ASD group in the right temporoparietal junction (BA39); left temporoparietal junction (BA39); posterior cingulate cortex (BA31); and the dorsomedial prefrontal cortex (BA10) (Pokorny et al., 2018; see Table 3 and Figure 4). In Fourie et al. (2020) the whole-brain analyses contrasting all (functional and communicative) gestures combined vs. the undefined baseline condition showed no significant group differences in brain activation.

##### Contrast effects in analyses of different observational conditions

Comparison of the fully visible transitive vs. intransitive conditions (TD > ASD) showed no significant group differences, and age did not affect these outcomes (Pokorny et al., 2015). Similarly, whole-brain analyses comparing communicative > functional gestures yielded no significant effects between groups (TD > ASD; ASD > TD), neither did group comparisons contrasting the three movement intensity levels, or analyses of the three specified ROIs using three-way ANOVAS (movement type × movement intensity × group; Fourie et al., 2020). Likewise, whole-brain comparisons of activation related to observing action clips of a hand interacting with objects did not yield any significant between-group effects (Knaus et al., 2023), neither did analyses of the number of active voxels in the identified ROIs (the bilateral POP and SMG) in the same study.

##### Associations between behavioral measures and brain activity

Pokorny et al. (2018) examined associations between behavioral outcomes from the DCDQ with mean parameter estimates (beta) from each ROI by averaging the parameter estimates of all voxels in each region and found no significant associations.

### Synthesis of results

#### Overlapping activity and summary of group differences

Figures 2–4 show illustrative summaries of the between-groups effects found in the included studies. Some brain areas had activation differences between the ASD and TD groups in more than one study domain. For action execution and imitation, these areas included parts of the cerebellum; specifically, right posterior cerebellar activation (TD > ASD) was seen in both execution (Mostofsky et al., 2009) and imitation (Jack and Morris, 2014) whereas left posterior cerebellum (TD > ASD) was reported for imitation (Jack and Morris, 2014) and left anterior cerebellum (TD > ASD) for execution (Mostofsky et al., 2009).

For observation and imitation, there were activation differences in the cingulate cortex, however in different directions and hemispheres: observation in the right BA31 (TD > ASD, Pokorny et al., 2018) and for imitation in the right BA32 (ASD > TD, Williams et al., 2006) and left BA24 (TD > ASD, Wadsworth et al., 2017). Also, there were activation differences in the IPL (all TD > ASD) for observation (bilateral BA39, Pokorny et al., 2018) and imitation (right BA39, Wadsworth et al., 2017; bilateral BA40, Williams et al., 2006).

For observation only, a group difference (TD > ASD) was found in the right dorsomedial prefrontal cortex (BA10, Pokorny et al., 2018). For imitation only, there were group differences (TD > ASD) reported by Wadsworth et al. (2017) in the right IFG (BA44); and in both directions by Williams et al. (2006): TD > ASD in right fusiform and middle temporal gyri (BA37), ASD > TD in bilateral temporal regions (right BA38, left BA21), right parahippocampal gyrus (BA28), left M1 and PMC (BA4 and BA6), left uncus/amygdala, and the left claustrum.

#### Within-group effects

Presented below is available information about within-group activations reported by all included articles in the areas where between-group differences were found. Since these results are not direct group comparisons, we here chose to also involve ASD sub-group results (ASD low language ability, ASD high language ability) reported by Knaus et al. (2023). Within-group results (experimental condition > baseline) were not included in two of the studies (Jack and Morris, 2014; Williams et al., 2006); only for ASD (sub-groups) but not TD in one study (Knaus et al., 2023); and only presented for the two masks not yielding between-group differences in one study (AON, STS; Pokorny et al., 2018). One study presented effect sizes but not cluster sizes (Pokorny et al., 2015).

##### Premotor cortex (BA6)

Mostofsky et al. (2009) found activation from action execution in an area of left BA6 for TD (ipsilaterally, *k* = 174, *Z* = 8.35) but not ASD, as well as activation in a bilateral BA6 area with left-sided cluster peaks, seen contra- and ipsilaterally for both groups (ASD contralaterally: *k* = 44, *Z* = 6.48; ASD ipsilaterally: *k =* 161, *Z* = 8.24; TD contralaterally: *k* = 231, Z = 9.58; TD ipsilaterally: *k* = 266, *Z* = 8.43). One observation study (Pokorny et al., 2015) reported significant within-group activation for TD in BA6 in one condition (visible intransitive, *Z* = 3.68) which was not seen in the ASD group. They also found left hemisphere activation around the inferior part of PMC/IFG area for both groups. The TD group’s activation peaked within BA6 for two observational conditions (visible transitive: *Z* = 4.37; visible intransitive: *Z* = 3.68) whereas the ASD group had comparable activation in both conditions, however with peaks located in the adjacent BA44. It can also be noted that the TD group had another activation in the left PMC ROI, with the peak however located in the adjacent BA8 according to our transformation (visible transitive: *Z* = 3.59). This activation was not seen in the ASD group. Pokorny et al. (2018) reported activation in left BA6 in the conventional placing condition for both TD (*k* = 23, *Z* = 3.18) and ASD (*k* = 59, *Z* = 3.44), and only for ASD in the unconventional placing condition (*k* = 19, *Z* = 2.48). Finally, the observation study by Fourie et al. (2020) reported within-group activation in left BA6 for the TD group (all gestures > baseline: *k* = 27, Z = 4.22) with no corresponding activity seen in the ASD group.

##### Occipital areas (BA18 and BA19)

Wadsworth et al. (2017) found activation in right BA19 during imitation for the ASD group (*k* = 3,992, *t* = 12.1) but not TD. Pokorny et al. (2018) found significant within-group activations in BA19 for both groups and all observational conditions in the masked analyses, where TD had larger cluster sizes than ASD in three of the conditions (conventional eating, TD: *k* = 707, *Z* = 5.27; conventional eating, ASD: *k* = 114, *Z* = 4.37; unconventional eating, TD: *k* = 727, *Z* = 4.65; unconventional eating, ASD: *k* = 155, *Z* = 4.64; conventional placing, TD: *k* = 487, *Z* = 4.36; conventional placing, ASD: *k* = 345, *Z* = 5.30), whereas ASD showed a larger cluster of activation in one condition (unconventional placing, TD: *k* = 558, *Z =* 5.16; unconventional placing, ASD: *k* = 645, *Z* = 5.59). The TD group had an additional significant activation in the right BA19 (unconventional placing: *k* = 618, *Z* = 4.69) which was not seen in ASD. Knaus et al. (2023) found activation in the left BA19 in the low language ability sub-group during action observation (*k* = 4,590, *Z* = 4.9), not present in the high language ability sub-group.

##### Temporal areas (BA37)

In the masked analysis focusing on the STS, Pokorny et al. (2018) found activation in the right BA37 for the ASD group in all observational conditions. The TD group had fewer, but larger, activations peaking in right BA37. Both groups showed significant activations in this region in the conventional eating condition (TD: *k* = 1,007, *Z* = 5.11; ASD: *k* = 122, *Z* = 4.59) and the conventional placing condition (TD: *k* = 665, *Z* = 4.49; ASD: *k* = 258, *Z* = 4.41). The unconventional eating condition showed significant activation in the ASD group (*k* = 103, *Z* = 4.12) but not in the TD group, likewise for the unconventional placing condition (ASD: *k* = 716, *Z* = 4.66). Knaus et al. (2023) found significant activation in this area during action viewing in the low language ability group (*k* = 5,468, *Z* = 4.51) but not in the high language ability group.

##### Inferior parietal lobule (BA39 and BA40)

Mostofsky et al. (2009) found significant activation within left BA40 in the TD group for left-hand execution (*k* = 163, *t* = 7.79) and no corresponding activation in the ASD group. Wadsworth et al. (2017) reported significant left-side BA40 activation for the ASD group during imitation (*k* = 1,503, *t* = 9.3). The TD group did not have a comparable activation; they did however have a significant activation in the right IPL which peaked in BA7 (according to our transformation from MNI to BA). The two studies by Pokorny et al. (2015, 2018) found several activations in BA40 for both groups, where one study found overall more activations in the TD group (Pokorny et al., 2015) and the other in the ASD group (Pokorny et al., 2018). In Pokorny et al. (2015), both groups had three clusters of activation in bilateral BA40 in the visible transitive condition (TD *Z* = 4.90, 4.50, 2.91; ASD *Z* = 4.63, 4.16, 3.78). In the visible intransitive condition, TD had one left-sided and one right-sided cluster of activation (*Z* = 5.55 and 3.07, respectively) whereas ASD had one active cluster in the left hemisphere (*Z* = 3.61). The hidden transitive condition yielded one left-sided cluster of activation in TD (*Z* = 3.77) and two left-sided clusters in ASD (*Z* = 4.52, 4.29). The hidden intransitive condition showed two right-sided and two left-sided clusters of activation in the TD group (*Z* for right hemisphere clusters = 3.14, 2.89; *Z* for left hemisphere clusters = 4.20, 3.48) whereas the ASD group had two left hemisphere clusters of activation (*Z* = 3.50, 3.24). Summarizing the findings in Pokorny et al. (2018) of both placing conditions (conventional and unconventional placing) in the AON mask, a larger number of active clusters were found in BA40 for the ASD (six in total) than TD group (two in total, one left- and one right-sided), active clusters were generally larger for ASD (*k* ranging between 212 and 286, mean *k* for all six clusters = 252) than TD (*k* = 149 and 253), and activations were generally stronger in ASD (*Z* ranging between 3.2 and 4.46; mean *Z* of 3.9 for all six clusters) than TD (*Z* = 3.82 and 3.78). The STS mask in the same study yielded one activation for TD in the left BA39 (unconventional placing condition, *k* = 26, *Z* = 3.37).

##### Inferior frontal gyrus (BA44)

Pokorny et al. (2015) found activation in the right IFG in TD for all four conditions (visible transitive, *Z* = 4.80; visible intransitive, *Z* = 4.40; hidden transitive, *Z* = 3.46; hidden intransitive; 3.85), and for ASD in three conditions (visible transitive: *Z* = 3.49; hidden transitive: *Z* = 3.63; hidden intransitive: *Z* = 4.09). In the AON mask analysis, Pokorny et al. (2018) found activation for the ASD group in the conventional placing condition (*k* = 81, *Z* = 3.25) and for both groups in the unconventional placing condition (TD: *k* = 183, *Z* = 3.15; ASD: *k* = 59, *Z* = 3.39). Knaus et al. (2023) found significant within-group activation in the high language ability group in right BA44 (*k* = 2,546, *Z* = 4.06) that was not seen in the low language ability group.

##### Cerebellum

During action execution, both groups had significant activation in parts of the cerebellum (Mostofsky et al., 2009). Right-hand execution yielded activation in the right posterior/inferior cerebellum (Lobule VIII A/B) in the TD group (*k* = 228, *t* = 16.66). Both groups had significant activation in the left anterior cerebellum (lobules IV/V) during left-hand execution (TD: *k* = 760, *t* = 19.01; ASD: *k* = 264, *t* = 10.76), and the TD group had activation in the same area also during right-hand execution (*k* = 188, *t* = 9.48). The TD group also showed significant activation in the right posterior cerebellum (lobule VI) during left-hand execution (*k* = 94, *t* = 8.49), not seen in the ASD group.

## Discussion

The aim of this systematic review was to summarize the current knowledge of brain activation from fMRI studies focusing on execution, imitation, and observation of naturalistic actions in autistic children and adolescents. In keeping with our expectations, we found recruitment of largely similar brain regions in both groups, although with significant group differences in terms of magnitude and direction. This is in line with the accumulated knowledge from resting-state (rs) fMRI studies reporting neural under- and overconnectivity in ASD groups of children, adolescents and adults when compared to TD/NT groups (Hull et al., 2017).

Several of the included studies implicated atypical cerebellar activity in the ASD group. For example, the one study included in this review focusing on action execution (Mostofsky et al., 2009) found significant group differences (TD > ASD) in several parts of the cerebellum, and differing activation patterns when investigating cluster- and effect sizes in each group. For instance, activity in the right posterior cerebellum differed significantly in the group comparison. This area was also found to be active in the TD group (task > rest) during both ipsilateral and contralateral hand execution, whereas no significant within-group activation was found in the ASD group. Interestingly, studies succeeding Mostofsky et al. (2009) have provided further evidence that cerebellar abnormalities (structural, functional, and in connectivity) in the right posterior cerebellum are related to more severe ASD impairments in several domains, for example movement, language and social processing (D’Mello and Stoodley, 2015; Lidstone et al., 2021). It can also be noted that both groups had within-group activation in the right anterior cerebellum (however no significant difference between groups) during right-hand execution, where the TD group’s activation had a larger volume (1,041 vs. 206 voxels) and higher *t*-value (15.62 vs. 7.59) than ASD (Mostofsky et al., 2009). Moreover, findings of decreased motor circuitry (cerebellum/thalamus and SMA) connectivity were reported. Since cerebellum is highly involved in the formation and use of internal models (Wolpert et al., 1998; Streng et al., 2022), the findings from Mostofsky et al. (2009) support the idea of atypical use of internal action models as a common basis for the autistic phenotype (Mostofsky and Ewen, 2011).

Between-group differences in cerebellar activity were also found during action imitation (Jack and Morris, 2014). Furthermore, Jack and Morris (2014) found that stronger connectivity (higher PPI values) between the right crus I and the right posterior superior temporal sulcus (RpSTS) predicted higher mentalizing skills (ToMI scores) in the ASD group. Recruitment of the RpSTS alone did however not predict mentalizing skills, indicating the importance of connections between cerebellar and distal cortical brain areas such as the STS for understanding of others’ mental states. In addition, Wadsworth et al. (2017) found a significant within-group activation for TD in the right posterior cerebellum (i.e., a cerebellar area outside of our areas of interest) in the imitation vs. baseline whole-brain analysis, whereas the ASD group did not show any significant activation in the cerebellum. Taken together, the atypical activations found are consistent with theories of aberrant development and use of internal action models in autism (Mostofsky and Ewen, 2011). Atypical cerebellar activity in autistic individuals also seems to be persistent in adulthood (Stillesjö et al., 2025).

Other domain-overlapping activity found between the included studies were shared by observation and imitation studies and located in the cingulate cortex (right side: Pokorny et al., 2018; Williams et al., 2006; left side: Wadsworth et al., 2017) and in the IPL (bilaterally: Pokorny et al., 2018; Williams et al., 2006; right side: Wadsworth et al., 2017). The cingulate cortex is functionally heterogeneous, but its activation during action observation and imitation may be related to its importance for continuous performance monitoring and error detection (Carter et al., 1998; Säfström and Domellöf, 2018). The IPL (particularly BA40) is a core node in the AON and maps sensory representations to motor plans, supporting imitation and the understanding of goal-directed actions during observation (Caspers et al., 2010). Notably, all of these functions can be related to motor representation activity in terms of action performance and observation based on stored motor representations, and refinement of internal action models. Thus, the atypical engagement in cingulate and parietal cortices found in young autistic individuals further supports a difference in the formation and use of motor representations during development.

Against our expectations, reports of group differences in the IFG were scarce. Functional activation in this area has been shown to differ between autistic and neurotypical adults (Stillesjö et al., 2025) but thus appear more similar between autistic and TD children/youth. Still, one of the imitation studies did report significant group differences in activation of the IFG (Wadsworth et al., 2017) and it cannot be ruled out that a larger body of research would reveal atypical IFG activation also in young autistic individuals. Notably, a recent study using functional near-infrared spectroscopy (fNIRS) to investigate cortical activation related to observation, production, and imitation of communicative gestures in ASD vs. TD children found hyperactivation of IFG for the ASD group during imitation and observation (Su et al., 2023), supporting IFG involvement also during development. Alternatively, the previously observed differences in IFG activation between autistic and neurotypical adults could be due to developmental aspects of sensorimotor functioning that may be more evident in adults compared to children.

While several studies included measures of general motor skill, few associated such outcomes with imaging data. Still, reported outcomes mainly highlighted less proficiency in autistic compared with neurotypical children, which would also be expected given the reported differences in neural activity patterns.

Although a majority of study participants were male, the included studies had more equal sex distribution than that reported for adult ASD and TD groups in our previously published review (Stillesjö et al., 2025). This allows better generalizability to a female presentation of autism, even though the limited number of studies still makes generalization problematic. The included studies had equal groups in terms of IQ, or they controlled for group differences by adding intelligence measures (FSIQ or subscales) as regressors. Overall, IQ levels were relatively similar between studies as well. Therefore, IQ levels were not a confounding factor when comparing and synthesizing the studies’ results. It should however be noted that these results are difficult to translate to autistic youth with intellectual disability and/or more severe autism. Interestingly, Knaus et al. (2023) found significant group differences in brain activation when comparing the two sub-groups of the ASD population (low vs. high language ability) but not in the TD vs. ASD group comparisons. Also, Wadsworth et al. (2017) reported a correlation where, independent of group (TD, ASD), milder symptom severity correlated with higher activation in the right IPL. Both these findings suggest that autistic individuals with higher levels of symptom severity may show different patterns of activation than so-called “high functioning” autistic individuals, underlining the importance of including various ASD sub-groups in research.

### Limitations

While the risk of bias outcome was deemed low, the results reported in this review must be interpreted with caution given several factors that limit the feasibility of a more precise synthesis of findings. First, the number of studies included is limited, and the sample sizes are small. Second, various experimental tasks have been used in the studies, with different choices of stimuli and contrasts between conditions, and the reported results may thus partly be task specific. Also, there is substantial variability between the studies regarding additional methodological and design-related issues, such as pulse sequences, preprocessing choices, voxel size, spatial templates, statistical thresholds, and correction methods. In addition, the studies used different coordinate systems (MNI, Talairach). These systems are broadly comparable, but small differences may hamper the anatomical alignment of reported activations. Finally, although the Brodmann cytoarchitectonic map is useful for synthesizing results reported in different systems, it is a coarse way of mapping out fMRI results. Further, when conducting a systematic review it is always possible that an alternative search strategy in terms of, e.g., search string could render a different search outcome. Since the scope of this systematic review is narrow, and involves research that is associated with methodological challenges, a low number of included studies was to be expected and we believe that we have found the relevant sources available to date.

## Conclusion and future directions

There are currently only a limited number of fMRI-studies that have investigated naturalistic motor behavior (action execution, imitation and observation) in autistic children and adolescents. Since motor difficulties are prominent in autism, further research is warranted to increase the understanding of atypical motor processing in developing autistic individuals, and how it may be related to symptoms and neural processing in other domains. While fMRI is the functional brain imaging method with the best spatial resolution (cortically and subcortically), fNIRS allows for more ecologically valid paradigms when studying motor processing (although at the limit of subcortical and cerebellar activity) and, further, is commonly a suitable method for participants that are not eligible for fMRI. More research using both methods is thus needed to get a more complete understanding of atypical motor processing in autism. Ideally, studies should be designed to combine several imaging techniques and/or behavioral measures. Further, to facilitate future synthesis of research, we recommend transparent reporting of methods (in terms of, e.g., preprocessing steps, experimental conditions and contrasts) and results (e.g., including results from whole-brain analyses even if specific masked analyses are of main interest).

The body of evidence so far suggests atypical activity primarily in the cerebellum (action execution and imitation), the cingulate cortex (action imitation and observation), and the IPL (action imitation and observation) in autistic youth. These findings harmonize with the proposal of atypical establishment and employment of motor representations in autism during development. Further investigations into this potential relationship are of clinical and educational importance, as it may affect various domains such as motor performance, social behavior, and observational learning.

## Data Availability

The original contributions presented in the study are included in the article/Supplementary material, further inquiries can be directed to the corresponding author/s.

## Footnotes

1
https://bioimagesuiteweb.github.io/webapp/mni2tal.html
