# Supplementary material for: Execution, imitation and observation of naturalistic actions in autistic children and adolescents: a systematic review of fMRI studies
Source: Front Hum Neurosci. 2026 Apr 13;20:1786807. doi: 10.3389/fnhum.2026.1786807 (PMC13111386; doi:10.3389/fnhum.2026.1786807)
Supplement: Supplementary file 1 [file Table_1.docx]

Table S1: *Risk of bias assessment*

|  |  |  | **Study** | | | | | | | |
| --- | --- | --- | --- | --- | --- | --- | --- | --- | --- | --- |
| **Evaluation aspect** | | **Scoring** | Fourie et al. 2020 | Jack & Morris 2014 | Knaus et al. 2023 | Mostofsky et al. 2009 | Pokorny et al. 2018 | Pokorny et al. 2015 | Wadsworth et al. 2017 | Williams et al. 2006 |
| ***Sample characteristics*** | |  |  |  |  |  |  |  |  |  |
|  | Participants with autism were recruited based on their diagnosis | Yes = 1; No = 0 | 1 | 1 | 1 | 1 | 1 | 1 | 1 | 1 |
|  | Autism diagnosis was verified for the participants in the target group using specific standardized tests | For all participants = 2; For more than half the group = 1; For less than half the group = 0; | 2 | 2 | 2 | 2 | 1 | 1 | 2 | 2 |
|  | Age is reported with central tendency and spread (mean or median and standard devation or range) and distribution of sex is reported | Yes = 2; The distribution of sex *or* age is reported adequately = 1; No = 0 | 2 | 2 | 2 | 2 | 2 | 2 | 2 | 2 |
|  | Control subjects were recruited according to inclusion criteria, or evaluated to exclude psychiatric and medical illnesses, or presence of psychiatric and medical illnesses was used as exclusion criteria | Yes = 1; No or not described adequately = 0 | 1 | 1 | 1 | 1 | 1 | 1 | 1 | 1 |
|  | Important clinical variables of comorbidities and medication are described | Yes = 1; No or not described adequately = 0 | 0 | 1 | 0 | 1 | 0 | 0 | 1 | 1 |
| ***Recruitment/ selection bias*** | |  |  |  |  |  |  |  |  |  |
|  | A sampling strategy/method was used to ensure representativeness to the autism population considered | A defined procedure for recruitment and inclusion is specified = 1; No information is presented = 0 | 0 | 0 | 0 | 0 | 0 | 0 | 0 | 0 |
|  | Recruitment of autism participants | No selection bias was introduced = 1; Recruitment process not described adequately for evaluation of selection bias, or a specific selection bias was introduced = 0 | 0 | 0 | 0 | 1 | 0 | 0 | 0 | 0 |
|  | Recruitment of control participants | Controls were chosen from the same community as individuals with ASD (e.g., same samples of students/class/club) = 2; Control subjects were chosen from different populations (e.g., different schools or settings) however within the same wider community = 1; Information was unclear or not provided = 0 | 2 | 0 | 1 | 1 | 0 | 0 | 1 | 1 |
|  |  |  |  |  |  |  |  |  |  |  |
| ***Reproducability/ comparibility*** | |  |  |  |  |  |  |  |  |  |
|  | Comparability of individuals between groups | Groups are comparable according to sample size/composition (e.g., sex, age) where comparability is statistically controlled or groups matched = 1; Significant differences exist or groups are not matched = 0 | 1 | 1 | 1 | 1 | 1 | 1 | 1 | 1 |
|  | Measures are taken to control for IQ as a possible confounding factor | Groups are comparable on at least one IQ outcome, or group differences in IQ are controlled statistically in the fMRI analyses = 1; IQ outcomes are not reported, or significant group differences are not accounted for in fMRI analyses = 0 | 1 | 1 | 1 | 1 | 1 | 1 | 1 | 1 |
|  | Sub-group comparisons are made or described on demographic variables if only a sub-sample of the original sample is included in the fMRI analyses | A sub-sample exists and is compared to the control group on important demographic outcomes or a sub-sample does not exist = 1; A sub-sample exists but is not described nor compared on important demographic outcomes = 0 | 0 | 1 | 1 | 1 | 1 | 1 | 1 | 1 |
| ***Methodology and reporting*** | |  |  |  |  |  |  |  |  |  |
|  | Groups underwent the same experimental fmri procedure | Yes = 1; No or not described = 0 | 1 | 1 | 1 | 1 | 1 | 1 | 1 | 1 |
|  | Drop-outs are described | Yes or clearly does not exist = 1; No = 0 | 1 | 0 | 0 | 0 | 1 | 1 | 0 | 0 |
|  | Sample size | Justified and satisfactory method for defining sample size is described (e.g., power analysis) and size is appropriate = 2; Justified but not satisfactory or not justified but satisfactory (e.g., large sample) = 1; Not justified (none of the above conditions met) = 0 | 1 | 1 | 1 | 1 | 1 | 1 | 1 | 1 |
|  | Outcomes are clearly described so that they can be reproduced (e.g., beta values, voxel sizes) | Yes = 1; No = 0 | 1 | 1 | 1 | 1 | 1 | 1 | 1 | 1 |
|  | Whole brain analysis was automated with no a-priori regional selection | Yes = 1; No = 0 | 1 | 1 | 1 | 1 | 0 | 0 | 1 | 1 |
|  | Magnet strength was at least 1.5T | Yes = 1; No = 0 | 1 | 1 | 1 | 1 | 1 | 1 | 1 | 1 |
|  | Slice thickness <= 1 mm on structural T1 images and <= 4 mm on functional MRI images | Yes = 1; No = 0 | 1 | 0 | 1 | 0 | 1 | 1 | 0 | 0 |
|  | Acquisition and preprocessing techniques were clearly described so that they could be reproduced | Yes = 1; No = 0 | 1 | 1 | 1 | 1 | 1 | 1 | 1 | 1 |
|  | A sufficient cluster-forming threshold was used (i.e., voxel-based uncorrected p-value) to compensate for false positive underestimation | Yes = 1; No = 0 | 1 | 1 | 1 | 1 | 1 | 1 | 1 | 0 |
|  | Significant differences are reported in a standard space | Yes = 1; No = 0 | 1 | 1 | 1 | 1 | 1 | 1 | 1 | 1 |
|  | Significant results were reported after correction for multiple testing using a standard statistical procedure (FDR, FWE or permutation-based methods) | Yes = 1; No = 0 | 1 | 1 | 1 | 1 | 1 | 1 | 1 | 0 |
|  | Statistical tests are clearly described and appropriate, and results are clearly presented | Yes = 1; No = 0 | 1 | 1 | 1 | 1 | 1 | 1 | 1 | 1 |
|  | Conclusions are consistent with the results obtained and limitations are discussed | Yes = 1; No = 0 | 1 | 0 | 1 | 1 | 0 | 1 | 1 | 1 |
|  | A baseline was used and clearly defined | Yes = 1; No = 0 | 0 | 0 | 0 | 1 | 1 | 0 | 1 | 1 |
|  | Comparison conditions/contrasts were clearly defined | Yes = 1; No = 0 | 1 | 1 | 0 | 1 | 1 | 1 | 1 | 1 |
| **Total score** | |  | **24** | **21** | **22** | **25** | **21** | **21** | **24** | **22** |
