# Supplementary material for: Execution, imitation and observation of naturalistic actions in autistic children and adolescents: a systematic review of fMRI studies
Source: Front Hum Neurosci. 2026 Apr 13;20:1786807. doi: 10.3389/fnhum.2026.1786807 (PMC13111386; doi:10.3389/fnhum.2026.1786807)
Supplement: Supplementary file 2 [file Table_2.docx]

| **Table S2** *fMRI parameters in all included studies* | | | | |  |  |  |  |  |  |  |  |  |
| --- | --- | --- | --- | --- | --- | --- | --- | --- | --- | --- | --- | --- | --- |
| **Study** | **Field** | **TR/TE (ms)** | **FA** | **Slices/ volume** | **Volumes** | **Slice Timing corr.** | **Motion corr** | **Nuisance reg.** | **FWHM (mm)** | **Norm. Template** | **Voxel size** | **Statistical Analysis** | **Band-pass filt.** |
| *Execution* |  |  |  |  |  |  |  |  |  |  |  |  |  |
| Mostofsky et al. 2009 | 1.5 T | 35/64 | 70 | 26 | N/A | Applied | N/A | Motion paramaters | Half the resolution of the design matrix (7x7x11 mm^3^) | Study-specific template to MNI space | 3.59 mm x 3.59 mm x 5.5 mm | Whole-brain: *p* = 0.0001, *k=*32 (uncorrected)  ROI: Bonferroni corrected significance threshold for ROI analyses:  *p <* 0.05, trends reported at  *p* < 0.1. | N/A |
| *Imitation* |  |  |  |  |  |  |  |  |  |  |  |  |  |
| Jack and Morris 2014 | 3 T | 2000/40 | 90 | 28 | 237 | Applied | > 3mm | N/A | 5 | MNI using FLIRT and FNIRT | 3.0 mm x 3.0 mm x 4.2 mm | Z > 2.3, k: p=0.05 (corrected) | High-pass temporal filter |
| Wadsworth et al. 2017 | 3 T | 1000/30 | 60 | 17 | 384 | Applied | N/A | N/A | 8 | MNI unspecified template | 3.75 mm x  3 .75x5 mm | *p* < 0.05 (FWE corrected), k> 30 (whole brain), k=20 (mask) | N/A |
| Williams et al. 2006 | 1.5 T | 3000/33 | 90 | 24 | N/A | Applied | > 5 mm | N/A | 8 | MNI standard template | 2 mm^3^ | *p* < 0.001 (uncorrected), k> 5 | N/A |
| *Observation* |  |  |  |  |  |  |  |  |  |  |  |  |  |
| Fourie et al. 2020 | 3 T | 2000/25 | 90 | 36 | N/A | Applied | N/A | Motion  parameters | 5 | MNI, avg152  T1-weighted  template | 3.4 mm^3^ | *p* < 0.05 (FWE), k> 20 | N/A |
| Knaus et al. 2023 | 3 T | 3000/30 | 90 | 31 | N/A | Fourier-space time-series phase-shifting, skull stripping of the T1 using BET | > 2 mm | Motion parameters | 6 | MNI 152  T1 template | N/A | Z > 2.3, p = 0.05 | High-pass temporal filter |
| Pokorny et al. 2015 | 3 T | 2000/30 | 90 | 32 | N/A | Cubic spline-timing interpolation to the  first non-discarded scan/run | > 3.4 mm |  | 5 | MNI standard  template  (nonspecified) | 3.4 mm^3^ | *p* < 0.005, k≥ 0 (FDR corrected) | High-pass temporal filter (128 sec) |
| Pokorny et al. 2018 | 3 T | 2000/30 | 90 | 32 | N/A | Cubic spline-timing interpolation to the  first non-discarded scan/run | > 3.4 mm | Movement parameters; signal differences across scanning runs | 5 | MNI standard  template  (nonspecified) | 3.4 mm^3^ | *p* < 0.05 (FDR corrected), k=10 | High-pass temporal filter (128 sec) |
|  |  |  |  |  |  |  |  |  |  |  |  |  |  |
